# Comparing Outcomes of Open and Robot-Assisted Inguinal Lymphadenectomy for the Treatment of cN2 Squamous Cell Carcinoma of the Penis: A Retrospective Single-Center Analysis

**DOI:** 10.3390/cancers16233921

**Published:** 2024-11-22

**Authors:** Aldo Brassetti, Rigoberto Pallares-Mendez, Alfredo M. Bove, Leonardo Misuraca, Umberto Anceschi, Gabriele Tuderti, Riccardo Mastroianni, Leslie C. Licari, Eugenio Bologna, Silvia Cartolano, Simone D’Annunzio, Mariaconsiglia Ferriero, Rocco S. Flammia, Flavia Proietti, Costantino Leonardo, Giuseppe Simone

**Affiliations:** Department of Urology, IRCCS “Regina Elena” National Cancer Institute, Via Elio Chianesi 43, 00143 Rome, Italy; aldo.brassetti@gmail.com (A.B.); leonardo.misuraca@ifo.it (L.M.); umberto.anceschi@ifo.it (U.A.); leslieclaire.licari@uniroma1.it (L.C.L.); costantino.leonardo@ifo.it (C.L.); giuseppe.simone@ifo.it (G.S.)

**Keywords:** penile cancer, penile neoplasms, lymph node excision, robotic surgical procedures, robot-assisted inguinal lymphadenectomy

## Abstract

Background: Inguinal lymph node (LN) dissection (iLND) is mandatory in cN2 penile squamous cell carcinoma (PSCC). Open iLND (OIL) is often omitted due to the high rate of complications. A minimally invasive approach may reduce morbidity; however, evidence supporting its role to treat bulky nodes is limited. This study aimed to present the outcomes of the largest European single-center series of robot-assisted iLND (RAIL) for the treatment of cN2 PSCC and to compare the surgical and survival outcomes of this approach with the standard of care. Methods: A retrospective analysis was conducted on men with cT1-4N2M0 PSCC undergone either OIL or RAIL at our institution from January 2014 onwards. Baseline demographics, perioperative data, and oncologic outcomes were analyzed. Results: Overall, 47 patients were included; 38 (81%) underwent OIL. Median age was 59 years, with 23 men (48%) presenting with a ≥4 Charlson comorbidity index. Operation time was significantly longer in the robotic cohort (212 min vs. 145 min; *p* < 0.001), while the length of stay (*p* = 0.09) and time to inguinal drainage removal (*p* = 0.08) were not. Estimated blood loss favored the robotic approach (60 mL vs. 300 mL; *p* < 0.001). Post-operative complications rates were comparable in the two groups (25% vs. 47%; *p* = 0.17): four major complications were observed overall, and these were all in the OIL cohort. Median LN yield was comparable between the two groups (18 vs. 25; *p* = 0.05). Final pathology reports showed no significant differences in tumor stage distribution between the cohorts (*p* = 0.54). Kaplan–Meier analysis did not reveal any significant differences in RFS probabilities between the two treatment groups (Log Rank = 0.99). Conclusions: RAIL demonstrated comparable perioperative and oncologic outcomes to OIL for cN2 PSCC, with the benefit of reduced estimated blood loss. RAIL is a feasible option for cases where a minimally invasive approach is preferred, offering comparable perioperative safety and oncological outcomes.

## Simple Summary

This study assessed 47 patients with penile cancer who underwent inguinal lymphadenectomy using either an open or robot-assisted approach. Our observations showed that robot-assisted inguinal lymphadenectomy resulted in longer operating times and less bleeding compared to the open approach. No significant differences were observed between the two groups for other perioperative variables. After a five-year follow-up, there were no differences in recurrence-free survival or overall survival between the groups.

## 1. Introduction

Penile cancer (PC) typically follows a predictable lymphatic spread, progressing to the inguinal lymph nodes (iLN), then involving the pelvic lymph nodes, and ultimately leading to distant metastases [1]. Radical inguinal lymph node dissection (iLND) has a therapeutic role in clinical N1–2 disease, but cN3 disease is rarely cured by surgery alone and requires multimodal management [2].

Despite the well-known impact of iLND on survival outcomes, it remains underutilized, probably because of the complication rate associated with the procedure, which ranges between 10% and 78% [3,4]. The interest in reducing complications associated with open iLND has naturally led to the exploration of minimally invasive approaches [2]. However, given the limited data available for patients with clinically positive nodes, the EAU-ASCO guidelines panel does not recommend the use of minimally invasive iLND techniques for cN1–2 patients outside of a clinical trial [5].

This retrospective single-center analysis aims to compare the perioperative and oncologic outcomes of open versus robot-assisted inguinal lymphadenectomy in patients with cN2 penile squamous cell carcinoma (PSCC).

## 2. Methodology

### 2.1. Patients and Methods

After obtaining institutional review board approval, we searched our prospectively maintained database for patients with cT1-4N2M0 PSCC who had undergone open (OIL) or robot-assisted iLND (RAIL) at our institution from January 2014 onwards. We conducted a comprehensive data collection process, gathering baseline demographic and anthropometric data (such as age, height, weight, and body mass index), information on patient comorbidities, as well as detailed perioperative (estimated blood loss (EBL), operation time (OT), intra-/post-operative complications (defined according to the Clavien–Dindo scale) [6] and oncologic outcomes. Additionally, we included final pathology findings in our analysis and assigned tumor stage according to the 8th edition of the American Joint Committee on Cancer (AJCC) classification system [7]. Patients with any missing data were carefully excluded to ensure the integrity of our results.

### 2.2. Interventions

At our institution, men presenting with PSCC and bulky iLNs nodes are routinely offered surgical treatment. Adjuvant systemic therapy is then proposed, based on histopathological findings. When feasible, the primary tumor is treated together with the iLNs. The decision for emasculation or partial penectomy is determined by the treating surgeon based on the local extent of the disease and patient preferences.

Radical iLND is offered to all patients with cT1-4N2M0 PSCC. The OIL is conventionally performed using the technique proposed by Yao, which involves dividing the fascia lata above the femoral vessels and en bloc removal of both superficial and deep lymph node packets while sparing the great saphenous vein when feasible [8].

All the robot-assisted procedures were performed by a single high-volume surgeon with extensive experience in minimally invasive surgery. The surgical team begins by identifying and marking the femoral triangle, which is defined by the inguinal ligament at the base, the adductor longus muscle medially, and the sartorius muscle laterally. An 8 mm camera port is then placed approximately 25 cm inferior to the midpoint of the inguinal ligament, just below the apex of the femoral triangle. Blunt dissection is performed using the index finger to develop the subcutaneous space in a cranial direction from the first port of entry. After the camera is inserted, the surgical field is insufflated with CO_2_ at a pressure of 10 mmHg. Two additional 8 mm robotic ports are placed starting from the camera port: one on the medial side, 8–10 cm in a slightly apical direction, and the other on the lateral side in a similar manner, positioned at the lateral boundaries of the femoral triangle. A 5 mm assistance port is positioned midway between the camera port and the lateral port. The robotic surgical platform is placed on the left side of the patient. The use of the Da Vinci Xi system eliminates the need for repositioning the platform when performing the re-docking for the contralateral inguinal lymph node dissection.

Both open and robotic surgery utilize a combination of non-absorbable clips, monopolar coagulation, and advanced sealing devices to achieve hemostasis and control lymphatic vessels.

An 18 Ch silicone drain is left after the surgery and removed once the output decreases to less than 100 mL per day.

All patients were offered a comprehensive lifelong follow-up program, with visits scheduled every 3 months for the first 2 years, followed by visits every 6 months until the 5th year, and then annually thereafter: cross-sectional imaging studies were performed in accordance with international guidelines.

### 2.3. Statistical Analysis

The study population was divided into two groups based on the surgical approach to iLND. Categorical variables were reported as frequencies and proportions and compared using the chi-squared test. Continuous variables were presented as medians and interquartile ranges and compared with the Mann–Whitney U test. Recurrence-free survival (RFS) analysis was conducted using Kaplan–Meier plots, and the log-rank test was used to compare the two groups. The significance level was set at a *p*-value of less than 0.05. Statistical analysis was performed using the Statistical Package for Social Sciences (Chicago, IL, USA) version 25.0.

## 3. Results

A total of 47 patients were included in the analysis, 38 of whom underwent OIL (81%). The median age at the time of surgery was 59 years (IQR: 53–69), with 23 men (48%) presenting with a Charlson comorbidity index (CCI) of 4 or higher.

Overall, 74% (*n* = 35) of the patients received a partial penectomy (either concurrently or shortly before inguinal lymphadenectomy), 74% in the OIL group vs. 78% in the RAIL group (*p* = 0.73). The remaining patients (*n* = 12) received a total (or subtotal) penectomy.

The baseline characteristics of the two groups were comparable, as shown in Table 1. The operative time (OT) was significantly longer in the robotic cohort (212 min vs. 145 min; *p* < 0.001), while the length of stay (LOS) (3 days vs. 3 days; *p* = 0.09) and time to inguinal drainage removal (28 days vs. 21 days; *p* = 0.08) were comparable in the two groups.

Estimated blood loss (EBL) favored the robotic approach, with an average of 60 mL compared to 300 mL in the open group (*p* < 0.001). Post-operative complications occurred in 16 (42.1%) and 4 (25%) patients in the OIL and RAIL groups, respectively, with the majority being Clavien–Dindo grade I (Table 2). Overall, four major complications were observed, all in the open cohort. These included three cases of lymphocele and minor wound dehiscence, managed in an outpatient setting under local anesthesia (Clavien–Dindo IIIa), and one case of severe wound dehiscence and lymphorrhea, requiring extensive debridement and the creation of a pedicled anterolateral thigh flap under general anesthesia (Clavien–Dindo IIIb).

The median lymph node yield was not significantly different between the two cohorts, with 25 nodes (IQR: 17–33) retrieved in the open group and 18 nodes (IQR: 12–24) in the robotic group (*p* = 0.05). Final pathology reports showed no significant differences in AJCC stage distribution between the cohorts (*p* = 0.54); only three patients presented with organ-confined disease, all of whom received OIL (8%).

**Figure 1:**
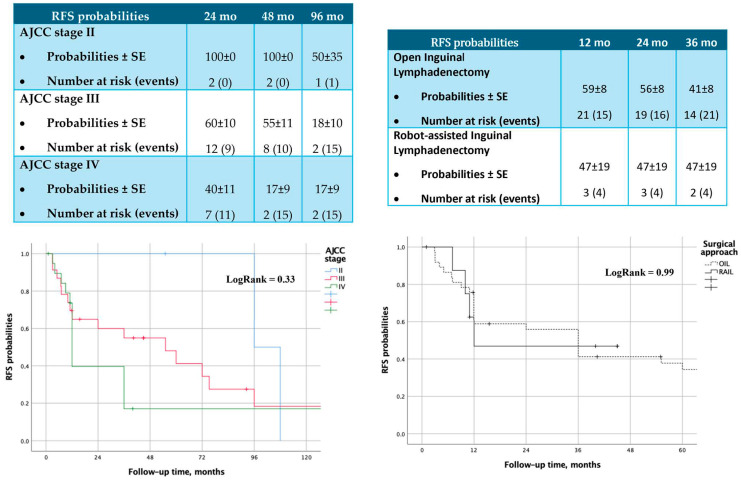
Recurrence-free survival probabilities, according to AJCC stages and surgical approaches.

**Figure 2:**
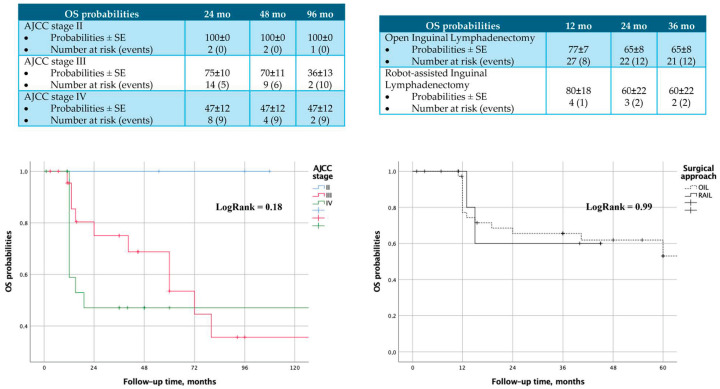
Overall survival probabilities, according to AJCC stages and surgical approaches.

Kaplan–Meier analysis did not reveal any significant differences in recurrence-free survival (RFS) probabilities between the two treatment groups (Log Rank = 0.99) (Figure 1 and Figure 2).

## 4. Discussion

PC is an aggressive and disfiguring disease, accounting for less than 0.1% of male cancers among western countries; a higher incidence can be observed in some regions, like India, South America, and Africa, where neonatal circumcision rates are lower [1]. Despite treatment, up to 25% of patients experience disease recurrence within the first year [9].

Similar to other squamous cell carcinomas, PC has a tendency for local and regional growth, and lymphatic spread typically occurs before hematogenous dissemination. Observations indicate that lymphatic drainage in these patients is often to both inguinal regions, occurring in up to 81% of cases, with metastatic cells following a consistent migratory pattern, as skip lesions and contralateral lymphatic metastases have never been reported [10]. The iLNs represent the primary drainage site, and subsequent metastatic progression typically involves the pelvic and then retroperitoneal nodes [11]. The iLNs are situated within the femoral triangle, a wedge-shaped area on the anteromedial aspect of the thigh, bordered by the inguinal ligament, the adductor longus, and the sartorius muscles. These nodes are topographically divided into superficial and deep compartments. The superficial nodes can be found between the Camper’s fascia and the fascia lata. The deep nodes lie below the fascia lata, medial to the femoral vein. Communications exist between the two compartments and eventually drain into the pelvic lymph nodes. Rouvière first introduced an inguinal lymph node division system into four quadrants, centered at the saphenous hiatus [12]. Later, Daseler added a fifth region, located at the saphenous-femoral junction [13]. More recently, Leijte et al. [14] highlighted that the superomedial quadrant contains most of the first draining nodes through lymphoscintigraphy, although variability between individuals may exist. Lymphovascular invasion, local stage, and tumor grade are known predictive factors for lymphatic metastases in penile squamous cell carcinoma. The risk of node-positive disease significantly increases with more advanced local tumor extension: approximately one-third of patients with T1 PSCC have lymph node metastases, while the rate doubles among those with locally advanced disease. The presence of lymph node involvement is clearly associated with poorer survival outcomes, as 3-year cancer-specific survival approaches 100% for pN0/N1 disease but drops to 73% for pN2 tumors [15].

Given the aggressive nature of this malignancy, prompt and appropriate treatment is essential to prevent rapid progression. The primary goal of surgery is to remove the primary tumor with clear margins while also resecting potential metastases from the groins of patients with high-risk disease or palpable inguinal lymphadenopathy [5]. Despite the clear impact of iLND on survival for patients with PSCC, significant underutilization of this procedure has been observed, likely due to its inherent morbidity. Two comprehensive retrospective studies reported adherence rates to iLND guidelines as low as 19.6–26.5% in men with PC [3].

OIL is the standard surgical treatment for patients diagnosed with high-risk PSCC. However, this procedure is associated with a considerable risk of complications, including local edema, seroma formation, wound dehiscence, tissue necrosis, deep vein thrombosis, and lymphoceles [16,17]. As a result, various surgical modifications have been proposed to mitigate these drawbacks and enhance the patient’s quality of life after surgery while maintaining the effectiveness of cancer treatment. Nevertheless, the complication rate remains unsatisfactorily high.

Given the success of minimally invasive approaches in reducing morbidity in other interventions, both conventional and robot-assisted laparoscopic iLND have also been used in patients with PC, yielding excellent outcomes. Singh et al. [18] compared 51 patients receiving RAIL to 100 patients undergoing OIL. The results of this study were comparable to ours; lymph node yield was similar between both groups (13 vs. 12.5 for RAIL and OIL, respectively), and major complications were less frequent in the RAIL group. Other variables, such as length of stay and days to drain removal, showed differences in this study. Furthermore, our results are consistent with other studies, which observed fewer complications and similar operating times [19]. Regarding some major complications, such as skin necrosis, open surgery jeopardizes blood supply through wide subcutaneous dissection and excision, which may also result in delayed healing in a considerable number of patients, that can go up to 50% [20,21]. In our study, no single skin necrosis or delayed wound healing was observed in the RAIL arm; furthermore, although statistical significance was not reached, Grade 3 Clavien–Dindo complications were observed only in the OIL arm. Other studies have reported no differences in complication rate or local recurrence [19]. Robot-assisted inguinal lymphadenectomy proves to be a non-inferior procedure compared to OIL. Importantly, several studies report fewer complications favoring RAIL [18,20,21,22]. Being minimally invasive, RAIL also promotes faster wound healing. Although no statistical significance was reached in our cohort, it is noteworthy that 26% of patients in the OIL arm experienced minor wound-related complications when compared to the RAIL arm (11%, *n* = 1).

Many technical variations in robotic approaches have been described (anterograde, retrograde, no-repositioning), though none have been directly compared in studies. A recent study, however, compared robotic and pure laparoscopic approaches, observing differences in operating time only (104 ± 13 vs. 136 ± 11 min for RAIL and pure laparoscopic, respectively) [23]. Our results are in line with the current bibliography [24]. Although our mean operating time was 271 min (216–299), docking was included in the time of surgery. Furthermore, median blood loss of the RAIL arm in our series was consistent with other previously reported studies [25]. While OIL may offer the advantage of reduced operating time, our study showed that RAIL, in contrast, provides other advantages such as shorter hospital stay, reduced bleeding risk, and fewer days to drain removal, as supported by other available evidence [18,24,25,26].

Regarding oncologic outcomes, our study has the strength of a long-term follow-up of 96 months. No significant differences in recurrence-free survival or overall survival were observed in our cohort across AJCC stages or surgical approaches at 24, 48, and 96 months. Tumor recurrence following inguinal lymphadenectomy for PC has been reported to range between 16–19% in patients with positive lymph nodes at 5 years of follow-up [27,28]. Our study showed comparable results at 96 months, with estimated recurrence probabilities of 50 ± 35%, 18 ± 10%, and 17 ± 9% for AJCC stage II–IV, respectively. Another study performed by Cozzi et al. [29] also reported long-term survival, though Kaplan–Meier analyses were not performed in their study. A large multicenter study assessing RAIL in three high-volume hospitals followed-up patients for an average of 13.5 months, reporting 13 recurrences and 10 cancer-related deaths; however, the study did not compare RAIL to other interventions [30]. Most studies to date show no differences between OIL and RAIL in terms of lymph node yield [18].

One limitation of RAIL is the high costs associated with the robotic system. To our knowledge, no studies have yet evaluated or compared the cost burden of RAIL versus OIL in the long term. It is important to interpret outcomes of minimally invasive procedures for inguinal lymph node dissection with caution, as much of the data comes from nonrandomized studies [24]. While robotic approaches offer greater dexterity in confined spaces, further prospective and randomized studies are needed to fully assess their value. Our study is limited by its retrospective design. Furthermore, we acknowledge that increasing the sample size would improve the reliability of our findings, making the results more robust and generalizable. Collaborating with other referral centers to gather additional data could further enhance the strength of our observations; nonetheless, we believe minimally invasive techniques to be adequate regarding oncological outcomes with a good risk–benefit balance. Due to the limited follow-up time in our study, further research with a longer follow-up period is needed to provide additional data on potential differences in the long-term impact of the oncologic outcomes measured. These outcomes are promising and warrant future investigations in a prospective matter.

## 5. Conclusions

Evidence concerning the robotic treatment of penile carcinomas with bulky inguinal lymph nodes is extremely limited, and guidelines recommend against its use outside of clinical trials.

According to our retrospective analysis, RAIL is as effective as OIL, allowing for the collection of a comparable number of nodes. The safety profile appears similar as well, given the comparable rates of overall complications. From an oncological perspective, the use of a minimally invasive approach does not seem to impact the probability of recurrence-free survival.

Although RAIL and OIL showed comparable outcomes in hospital stay, recovery time, complication rates, lymph node yield, and oncological outcomes, RAIL was associated with a lower risk of bleeding, suggesting it as a promising minimally invasive alternative for patients with cN2 penile cancer. Future collaborative studies are anticipated to further validate these findings.

As surgical techniques continue to evolve, the findings from this study may be further strengthened, with validation in key variables such as hospital stay, time to drain removal, lymph node yield, recurrence-free survival, and overall survival. Despite presenting an analysis conducted on the largest European series of RAIL for clinically node-positive squamous cell carcinoma of the penis, our data are not sufficient to draw definitive conclusions: multicenter studies on larger populations and within the context of randomized clinical trials will be necessary to demonstrate the non-inferiority of the robotic approach compared to the open technique.

## Figures and Tables

**Table 1:** Patients’ characteristics at baseline, according to surgical approach.

	Overall *n* = 47	OIL *n* = 38 (81%)	RAIL *n* = 9 (19%)	*p*
Age at surgery, years	59 (53–69)	58 (53–67)	68 (52–73)	0.23
BMI	26 (24.6–30.1)	25.9 (24.4–30.1)	26 (25.1–28.8)	0.65
Diabetes, *n* (%)	16 (34%)	13 (34%)	3 (33%)	0.96
Obesity, *n* (%)	12 (25%)	10 (26%)	2 (22%)	0.80
CCI, *n* (%)				0.36
0–1	0 (0%)	0 (0%)	0 (0%)	
2–3	24 (52%)	21 (55%)	3 (37%)	
≥4	23 (48%)	17 (85%)	6 (62%)	
cT, *n* (%)				0.72
Ta–T1	10 (21%)	9 (24%)	1 (11%)	
2	18 (38%)	14 (37%)	4 (44%)	
3	17 (36%)	13 (34%)	4 (44%)	
4	2 (4%)	2 (5%)	0 (0%)	

BMI = body mass index, CCI = Charlson’s comorbidity index, cT = clinical local tumor stage.

**Table 2:** Peri-/post-operative outcomes, according to surgical approach.

	OIL *n* = 38 (81%)	RAIL *n* = 9 (19%)	*p*
Saphenous vein sparing intent, *n* (%)	0 (0%)	6 (67%)	<0.001
OT, min	163 (123–180)	271 (216–299)	<0.001
OT to perform ILND	145 (115–180) *	212 (158–279) ^†^	0.04
EBL, mL	300 (250–350)	60 (55–100)	<0.001
LOS, d	3 (3–4)	3 (2–3)	0.09
Time to inguinal drainage removal, days	21 (21–30)	28 (24–32)	0.08
Intraoperative complications, *n* (%)	1 (3%)	0 (0%)	0.62
Post-operative complications, *n* (%)	18 (47%)	2 (22%)	0.17
Would-related complications	10 (26%)	1 (11%)	0.33
CD ≥ 3	4 (10%)	0 (0%)	0.31
LNs retrieved, median (25–75 IQR)	25 (17–33)	18 (12–24)	0.05
AJCC stages, *n* (%)			0.54
Stage II	3 (8%)	0 (0%)	
Stage IIIa–IIIb	20 (53%)	4 (44%)	
Stage IV	15 (39%)	5 (56%)	

* Based on data from 21/38 patients who did not undergo primary tumor tratment during the same surgical procedure. ^†^ Based on data from two out of nine patients who did not undergo primary tumor tratment during the same surgical procedure. OT = operation time, ILND = inguinal lymph nodes dissection, EBL = estimated blood loss, LOS = length of hospital stay, CD = Clavien–Dindo, LN = lymph nodes, AJCC = American Joint Committee on Cancer, IQR: Interquartile range.

## Data Availability

Data are available at: https://gbox.garr.it/garrbox/s/768JPhmhLZ8FWjF (accessed on 16 November 2024).
